# Progressive 3D biomedical image registration network based on deep self-calibration

**DOI:** 10.3389/fninf.2022.932879

**Published:** 2022-09-21

**Authors:** Rui Sun, Jun Wu, Yongchun Miao, Lei Ouyang, Lei Qu

**Affiliations:** ^1^Ministry of Education Key Laboratory of Intelligent Computing and Signal Processing, Information Materials and Intelligent Sensing Laboratory of Anhui Province, School of Electronics and Information Engineering, Anhui University, Hefei, China; ^2^Hefei Comprehensive National Science Center, Institute of Artificial Intelligence, Hefei, China; ^3^SEU-ALLEN Joint Center, Institute for Brain and Intelligence, Southeast University, Nanjing, China

**Keywords:** biomedical image registration, progressive registration, hierarchical registration, deep self-calibration, dynamic dataset augmentation

## Abstract

Three dimensional deformable image registration (DIR) is a key enabling technique in building digital neuronal atlases of the brain, which can model the local non-linear deformation between a pair of biomedical images and align the anatomical structures of different samples into one spatial coordinate system. And thus, the DIR is always conducted following a preprocessing of global linear registration to remove the large global deformations. However, imperfect preprocessing may leave some large non-linear deformations that cannot be handled well by existing DIR methods. The recently proposed cascaded registration network gives a primary solution to deal with such large non-linear deformations, but still suffers from loss of image details caused by continuous interpolation (information loss problem). In this article, a progressive image registration strategy based on deep self-calibration is proposed to deal with the large non-linear deformations without causing information loss and introducing additional parameters. More importantly, we also propose a novel hierarchical registration strategy to quickly achieve accurate multi-scale progressive registration. In addition, our method can implicitly and reasonably implement dynamic dataset augmentation. We have evaluated the proposed method on both optical and MRI image datasets with obtaining promising results, which demonstrate the superior performance of the proposed method over several other state-of-the-art approaches for deformable image registration.

## Introduction

The development of high-resolution light microscopy, sparse labeling techniques, neuronal tracking methods, and several advanced microscopic imaging pipelines have made it possible to map the entire mammalian brain at single-cell resolution, such as the fluorescence micro-optical sectioning tomography (fMOST), the light-sheet fluorescence microscopy (LSFM), and the serial two-photon tomography (STPT). However, those biological scans captured by different imaging pipelines at different locations or periods show certain differences in voxel intensity, image texture, and brain anatomy, which make it difficult to explore biological working mechanisms from multiple sources of information. In order to make full use of these precious data resources, it is important to align the anatomical structures of those scans into one coordinate system by image registration. In particular, deformable image registration (DIR) can handle local non-linear deformation of biological organs and has become an important technology in biomedical image processing and analysis.

A practical image registration generally includes a global linear registration and a local non-linear registration. The global linear registration always consists of a series of linear transformations, such as scaling, translation, and affine transformation, to achieve the alignment of the main structure of images. Then, the remaining local non-linear deformations are aligned by DIR algorithms. In the past few years, researchers have proposed many DIR methods. The traditional registration methods such as Elastix (Klein et al., 2010) and ANTs (Avants et al., 2009) aimed at optimizing a pair of images by continuous iteration and promoting the smoothness of the registration mapping relationship at the same time. They are effective and the registration task of various biological organs can be completed without training specific models. Recently, with the widespread application of deep learning in the field of computer vision, deep learning-based biomedical image registration algorithms have become a hot and attractive research direction (Litjens et al., 2017; Haskins et al., 2020). The current deep learning-based biomedical image registration algorithms can be divided into supervised learning methods and unsupervised learning methods on whether the training labels (ground truth) are required. The registration accuracy of supervised learning-based registration methods can be improved with the accuracies of supervised labels. However, labeling biomedical images is usually time-consuming and laborious, which promotes the development of unsupervised learning methods.

**Figure 1:**
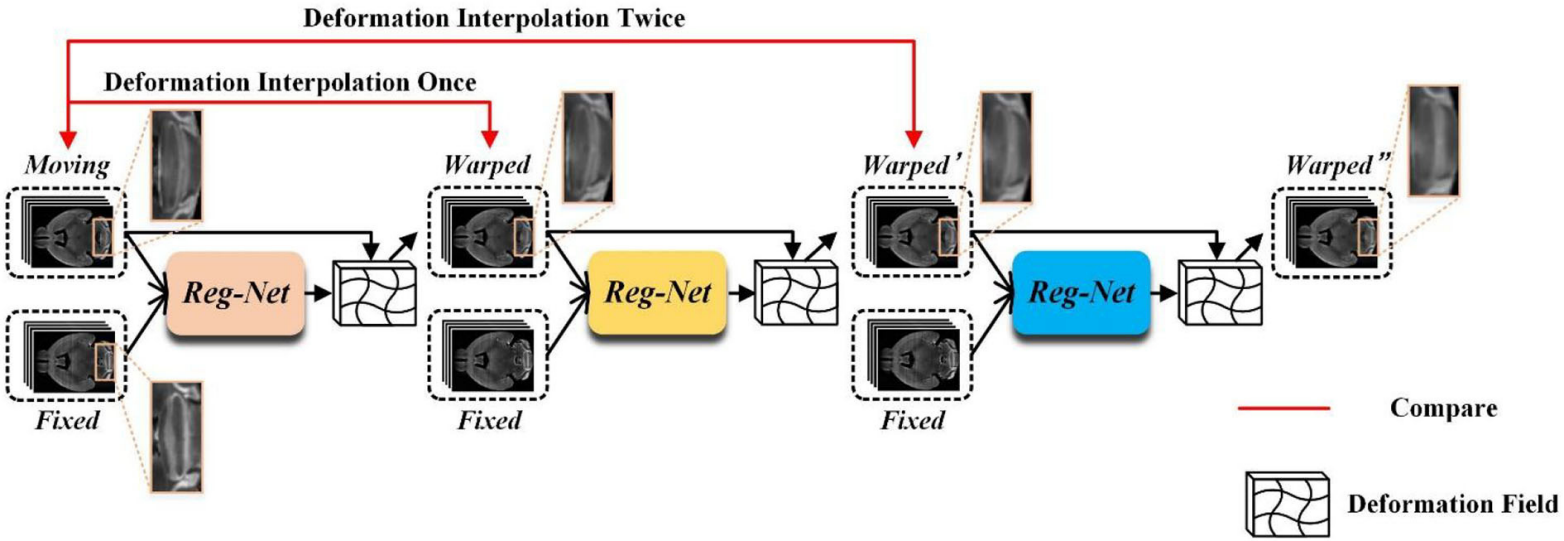
The example of three-stage cascaded network registration methods. Three different colored modules represent registration models with the same structure but no shared parameters.

Unsupervised registration methods always utilize the training data after global linear registration to learn the local non-linear deformation displacement vector field (Balakrishnan et al., 2018; Zhang, 2018; Qu et al., 2021), which usually focuses on describing small local deformations. Most of the existing methods cannot handle large deformation due to the smooth constraints imposed on the displacement vector field during the training phase. Although some cascaded registration methods (Cheng et al., 2019; Zhao et al., 2019a,b) and multi-scale registration methods (Kim et al., 2021; Shao et al., 2022) have been proposed to alleviate this issue, these still have some problems. For example, the multi-stage alignment process of cascaded methods may cause a serious information loss problem. Figure 1 shows the registration process of a three-stage cascaded registration network. The moving image is aligned onto the fixed image step by step through three registration networks by which the large deformation is transformed into three smaller ones. Although the large deformation problem is alleviated by this method, the information loss caused by interpolation is unavoidable at each stage of the warping process. As shown in Figure 1, as the number of interpolation increases, the structural boundary of the warped image in the orange box becomes more and more blurred. Furthermore, the multi-stage cascaded approach also tends to cause error accumulation. In addition, existing progressive registration methods (Cheng et al., 2019; Zhou et al., 2020; Kim et al., 2021; Zhang et al., 2021) integrate the displacement vector field by direct addition. However, the same position on the two adjacent displacement vector fields may not be the displacement of the same corresponding point, so it is unreasonable to obtain the total deformation field by directly summing the multiple deformation fields.

In this article, we aim to leverage the progressive registration idea of cascaded networks to deal with large deformations without causing information loss and increasing network parameters. Specifically, we propose a progressive registration network based on a deep self-calibration strategy. The main contributions of this article can be summarized as follows:

1. We propose a novel progressive registration strategy to reduce the cascaded information loss, in which an iterative registration strategy is implemented to decompose large deformation into small ones in multiple iterations. To avoid the cascaded information loss, we design a new displacement vector field integration algorithm to integrate the learned displacement vector field in each iteration into the total displacement vector field, and then the finally warped image can be generated with this total displacement vector field in one interpolation process.
2. We propose a new hierarchical registration strategy to achieve a fast and coarse-to-fine progressive registration to improve registration accuracy. The progressive registration strategy is implemented on a cascaded network with one low-resolution and one original-resolution network. With this hierarchical registration deployment, the images can be aligned on multiple scales to improve registration accuracy. Moreover, the low-resolution deformation field quickly learned by the low-resolution network can be upsampled and used as the initial value for the high-resolution network. Since the coarse-grained alignment of images is already done by the low-resolution network, using this deformation field can greatly improve the convergence speed of the high-resolution network.
3. The proposed progressive registration strategy can generate abundant training data during the training phase. By setting the number of iterations, the amount of training data generated can be controlled to achieve a dynamic data augmentation, thus effectively alleviating the limitations caused by the lack of training data.

The rest of the article is organized as follows. We review the related works about deformable biomedical image registration in Section Related works, followed by specifically introducing our proposed method and the detailed network structures in Section Method. Then the datasets, comparison methods, implementation, and evaluation metrics are described in Section Experiments configurations. The results are presented and analyzed in Section Results and analysis. Finally, Section Conclusion gives the conclusion of this work.

## Related works

### Traditional image registration methods

Traditional image registration methods here mainly refer to those non-learning-based algorithms, including intensity-based methods and landmark-based methods. Several popular non-learning-based methods have been designed for deformable registration such as elastic mapping (Bajcsy and Kovačič, 1989), Demons (Thirion, 1998), and HAMMER (Shen and Davatzikos, 2002). Diffeomorphic methods have also made a significant achievement in different computational tasks while preserving topology such as the large diffeomorphic distance metric mapping (Oishi et al., 2009; Zhang et al., 2017) and standard symmetric normalization (SyN) (Avants et al., 2008). Moreover, there were many existing biomedical registration pipelines such as the aMap (Niedworok et al., 2016), ClearMap (Renier et al., 2016), MIRACL (Goubran et al., 2019), ANTs (Avants et al., 2009), Elastix (Klein et al., 2010), and BIRDS (Wang et al., 2021).

Most of the abovementioned methods solve the registration task by iteratively exploring the space of potential transformation parameters based on a predefined objective function, which are always computationally intensive and time-consuming, and such characteristics also prevent these methods from being used in real-time clinical applications.

### Learning-based image registration methods

#### Supervised registration methods

Supervised learning-based registration methods need real or generated deformation fields as training labels to guide the network training. Dosovitskiy et al. (2015) proposed the FlowNet for 2D MRI brain registration, which utilizes statistical appearance models to generate ground truth to guide network training. Cao et al. (2017) extracted the patches of image pairs and used the spatial transformation relationship of corresponding patches generated by SyN (Avants et al., 2008) as labels to train the network. Ito and Ino (2018) utilized a convolutional neural network (CNN) to learn plausible deformations for ground truth generation. Fan et al. (2019) proposed the BIRNet which uses hierarchical and dual-supervised learning to predict the deformation field for 3D brain MR registration. Ni et al. (2021) proposed the DeepMapi by designing a sub-feedback strategy and a hierarchical registration strategy for 3D fMOST mouse brain registration.

While supervised learning methods have achieved considerable registration accuracy under the guidance of ground truth and high-quality synthetic labels, the difficulty in collecting label information greatly restricted their applications.

#### Unsupervised registration methods

The proposal of the spatial transformer network (STN) (Jaderberg et al., 2015) has led to the rapid development of unsupervised registration methods. Balakrishnan et al. (2018) proposed the VoxelMorph framework to implement a fast unsupervised end-to-end registration network. Zhao et al. (2019b) developed the VTN which achieves both end-to-end affine and non-rigid registration by cascading affine subnetworks. Zhao et al. (2019a) designed the recursive cascaded network which improves the registration performance by sequentially warping the image pairs through multiple cascaded sub-registration networks. Cheng et al. (2019) employed the U-Net cascaded separable convolution to complete large deformation and small deformation step by step. Zhang (2018) proposed the ICNet which adds the inverse-consistent constraint and anti-folding constraint to the loss function of the network in order to avoid local overlapping of deformation fields. Kim et al. (2021) devised CycleMorph which utilizes the topology consistency before and after registration to constrain the network and adopts the multi-scale and global-to-local registration strategy. Qu et al. (2021) designed the TIUNet to apply the inter-image deformation consistency constraint to network training.

Avants et al. (2008) declaimed that dice scores above 0.6 for smaller structures and 0.8 for larger structures are considered to be well-aligned. On this basis, we define the initial dice score of small structures in the data lower than 0.6 as large deformations and higher than 0.6 as small deformation. We call the initial dice score of larger structures in the data lower than 0.8 as large deformations and higher than 0.8 as small deformations. Existing unsupervised registration methods focus on aligning small local deformations between pairs of images. Therefore, these methods generally require global linear registration pre-processing to eliminate large linear deformations. However, there inevitably exist some large non-linear deformations in the pre-processed images due to imperfect pre-processing. Solving this large non-linear deformation is critical to improve the performance of the registration algorithm.

## Method

The goal of the learning-based unsupervised registration method is to estimate the non-linear transformation between two or more pairs of images without supervised information. The general objective function of the unsupervised registration method can be formulated in Equation (1),


(1) $$\begin{array}{l} L\left(M,F,DVF\right)=\;L_{sim}\left(\left(M\circ \phi \right),F\right)+\;\alpha^{\;*\;}L_{smooth}\left(DVF\right) \end{array}$$

where *M* and *F* represent the moving and fixed image, respectively. *DVF* indicates the displacement vector field and ϕ represents the deformation field. The relationship between DVF and ϕ is formulated in Equation (2),


(2) $$\begin{array}{l} \phi \left(x,y,z\right)=\left(x,y,z\right)+\;DVF\left(x,y,z\right) \end{array}$$

Additionally, *L*_*sim*_ represents the similarity loss between the warped image *M* ∘ ϕ and the fixed image *F*, and the smoothness constraint *L*_*smooth*_ is utilized to prevent the over-distortion of the area and too sharp displacement vector field. α is a weight parameter that controls the proportion of *L*_*smooth*_ in the total loss function. Since the smooth constraint is achieved by constraining the difference between adjacent points in the displacement vector field, it constrains the displacement between the large deformed anatomical region and its surrounding smooth region, thus limiting the ability of general one-shot deformable registration methods to perform large deformation registration.

**Figure 2:**
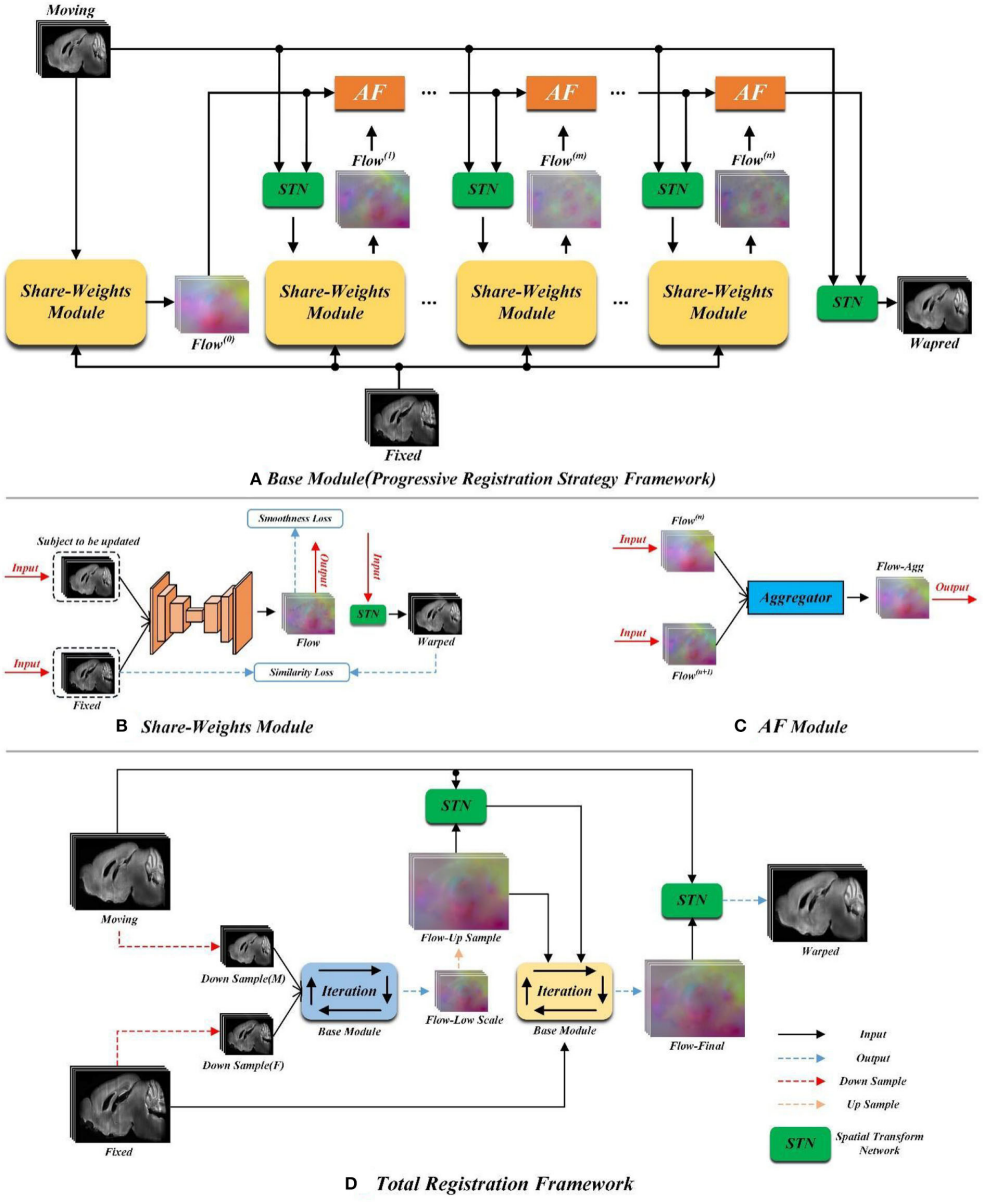
The pipeline of the proposed method. **(A)** Schematic diagram of the progressive registration strategy. **(B)** The structure of the yellow shared parameter module in **(A)**. **(C)** Schematic diagram of orange AF module in **(A)**. **(D)** Schematic diagram of total registration strategy.

The overview of our proposed method is shown in Figure 2. In order to solve the large non-linear deformation, we have designed three tailored registration strategies, including: (1) the progressive registration strategy, (2) the hierarchical registration strategy, and (3) the matching loss function. We will detail these novel designs in the rest of this section.

### Progressive registration strategy

Figure 2A demonstrates the progressive registration framework of the proposed method. A group of convolutional neural network (CNN) modules with shared weights are conducted to generate multi-stage deformation fields. Meanwhile, the aggregate flow (AF) module is designed to integrate multi-stage deformation fields into a total deformation field.

#### Shared weight module

**Figure 3:**
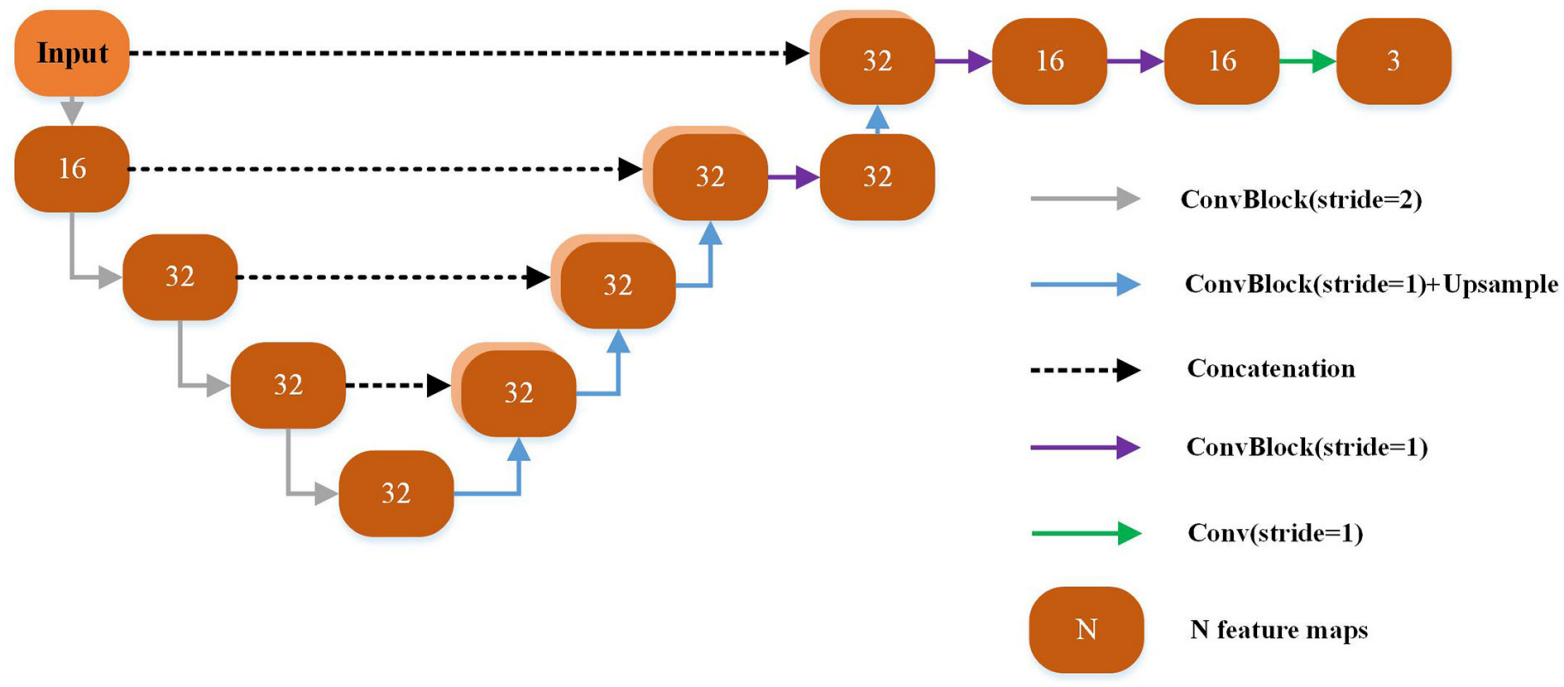
Overview of the CNN framework used in our proposed method.

Each yellow module in Figure 2A is a UNet-based registration module with shared parameters. The shared parameters are used iteratively in the non-training stage. The structure of the shared weight module is shown in Figure 2B. The displacement vector field between the input image pair can be learned by this module. The backbone of this module is a UNet as shown in Figure 3 which has been successfully applied to a variety of biological image segmentation and registration networks. The network consists of a decoder and an encoder with skip connections. In the encoder, each resolution stage has one *4* × *4* × *4* convolution layer, and the stride is set to *2* to down-sample the feature maps between each stage. In the decoder, each resolution stage has one upsampling layer and *3* × *3* × *3* convolution layer with a stride of 1. And the last two stages have two *3* × *3* × *3* convolution with a stride of 1 for finer estimation in detail. Each CONVblock is followed by a LeakyReLU. Finally, a *3* × *3* × *3* convolution without LeakyReLU is utilized to estimate a DVF with three channels for the deformation of each voxel in the x, y, and z-directions.

#### Aggregate flow module

Inspired by the invertibility loss formula in VTN (Zhao et al., 2019b), we propose the aggregate flow module. The detailed structure of the AF module is shown in Figure 2C. The inputs of the Aggregator in Figure 2C include *Flow*^(*n*)^ and *Flow*^(*n*+1)^(*n* ≥ 0) which stand for the displacement vector fields output by the network for two adjacent iterations. The function of the Aggregator is to integrate the displacement vector fields obtained from two adjacent iterations into a total displacement vector field. In the following formula, we use *Flow*_*Agg*^(*n, n*+1)^ to represent the total displacement vector field formed by integrating *Flow*^(*n*)^ and *Flow*^(*n*+1)^ with the Aggregator. And we adopt *F*(*x, y, z*) and *M*(*x, y, z*) to indicate the pixel point with the spatial coordinate of (*x, y, z*) on the fixed and moving image, respectively. *I* represents the displacement vector field (flow), and *I*(*x, y, z*) indicates the pixel point in the displacement vector field whose spatial coordinate is (*x, y, z*). ϕ represents the deformation field, and ϕ_*I*_*n*__ indicates the deformation field obtained by inputting *Flow*^(*n*)^ into STN. The symbol ∘ represents the warp operation, and *M* ∘ ϕ indicates the moving image warped by the deformation field ϕ. The aggregator can be expressed as (*I*_*n*+1_+(*I*_*n*_ ∘ ϕ_*n*+1_)), and its specific derivation process is detailed as follows.

Ideally, the moving image can be warped by the deformation field (ϕ) to obtain the same image as the fixed image as formulated in Equation (3),


(3) $$\begin{array}{l} F\left(x,y,z\right)=\left(M\circ \phi \right)\left(x,y,z\right). \end{array}$$

Combining Equations (2) and (3), we can obtain,


(4) $$\begin{array}{l} \;\left(M\circ \phi_{n}\right)\left(x,y,z\right)=M\left({\left(x,y,z\right)}^{{\;}^{\prime }}\right),\\ {\;(x,y,z)}^{{\;}^{\prime }}=\left(x,y,z\right)+I_{n}\left(x,y,z\right),\\ \left(M\circ \phi_{I_{n}}\right)\left(x,y,z\right)=M\left(\left(x,y,z\right)+I_{n}\left(x,y,z\right)\right). \end{array}$$

And from Equation (4), we can derive,


(5) $$\begin{array}{l} \;(\left(M\circ \phi_{I_{n}}\right)\circ \phi_{n+1})\left(x,y,z\right)\\ \;=\left(M\circ \phi_{I_{n}}\right)\left(\left(x,y,z\right)+I_{n+1}\left(x,y,z\right)\right)\\ =M\left[\left(x,y,z\right)+I_{n+1}\left(x,y,z\right)+I_{n}\left(\left(x,y,z\right)+I_{n+1}\left(x,y,z\right)\right)\right]. \end{array}$$

The combination of Equations (4) and (5) can finally be rewritten as Equation (6),


(6) $$\begin{array}{l} M\left[\left(x,y,z\right)+I_{n+1}\left(x,y,z\right)+\left(I_{n}\circ \phi_{I_{n+1}}\right)\left(x,y,z\right)\right]\\ =M\left[\left(x,y,z\right)+\left[I_{n+1}+\left(I_{n}\circ \phi_{I_{n+1}}\right)\right]\left(x,y,z\right)\right]\\ \;=M\circ \phi_{\left(\left[I_{n+1}+\left(I_{n}\circ \phi_{I_{n+1}}\right)\right]\left(x,y,z\right)\right)} \end{array}$$

Therefore, from the above formula derivation, the *Flow*_*Agg*^(*n, n*+1)^ can be equivalent to (*I*_*n*+1_+(*I*_*n*_∘ϕ_*n*+1_)).

The above formulae are extended to integrate the output *Flow* of *n* iterations of the network into a total displacement vector field *Flow*_*Agg*^(0, …, *n*)^, as shown in Equation (7),


(7) $$\begin{array}{r} {Flow\text{\_}Agg}^{\left(0,1\right)}=I_{1}+\left(I_{0}\circ \phi_{1}\right),\\ {Flow\text{\_}Agg}^{\left(0,1,2\right)}=I_{2}+\left({Flow\text{\_}Agg}^{\left(0,1\right)}\circ \phi_{2}\right),\\ \ldots \\ {Flow\text{\_}Agg}^{\left(0,\ldots ,n\right)}=I_{n}+\left({Flow\text{\_}Agg}^{\left(0,\ldots ,n-1\right)}\circ \phi_{n}\right). \end{array}$$

Therefore, the *Flow*_*Agg*^(0, …, *n*)^ can be gradually obtained with *n-1* AF Module integrations.

#### Elaboration of strategy

Figure 2A is the flow chart of our proposed progressive registration strategy. We iteratively use the registration module with shared parameters and complete the large displacement in space by multiple iterations without adding network parameters. The progressive registration strategy is detailed as follows.

Initial registration (zero iteration of the registration): Input the moving image and the fixed image into the shared weight module to generate *Flow*^(0)^. Then, the moving image and *Flow*^(0)^ are inputted into the STN to get the initial registration result, which is denoted as *Warped*^(0)^.

The first iterative registration: The *Warped*^(0)^ is utilized as the new moving image and inputted into the shared weight module with the fixed image again to generate *Flow*^(1)^. Then, the AF Module is conducted to integrate the displacement vector fields *Flow*^(0)^ and *Flow*^(1)^ to synthesize a total displacement vector field *Flow*^(0, 1)^. Subsequently, the moving image is warped to *Warped*^(0, 1)^ by the *Flow*^(0, 1)^.

The second iterative registration: The *Warped*^(0, 1)^ and the fixed image are inputted into the shared weight module again to obtain *Flow*^(2)^. Then, the *Flow*^(0)^, *Flow*^(1)^, and *Flow*^(2)^ are integrated into a total deformation field *Flow*^(0, 1, 2)^ by the Equation (7). Same as the previous iteration, the moving image is warped into *Warped*^(0, 1, 2)^ by the *Flow*^(0, 1, 2)^.

By extending the above procedure to a general form, we can obtain *Warped*^(0, …, *n*)^ by a total deformation field *Flow*^(0, …, *n*)^ in one interpolation process. We need to set the number of iterations of *n_train* and *n_test* of the network before the training and testing procedures which are independent of each other. The optimal numbers of *n_train* and *n_test* in this article are determined by comparing the experimental results.

### Hierarchical registration strategy

Figure 2D shows the overall registration architecture of our proposed method. We conduct two iterative registration networks with the same structure (as shown in Figure 2A) to build a cascade and multi-scale training. This multi-scale architecture has two advantages for fast and accuracy registration. First, the low-scale network has a larger field of perception, so it is capable of handling large deformations. Additionally, the smaller input size of the low-scale network leads to a smaller number of parameters, which could achieve coarse-grained fast registration. Second, we up-sample the deformation field generated from the low-scale network as the initial value of the original-scale network, which will facilitate the speed convergence of the original-scale network.

Specifically, as shown in Figure 2, the Down Sample (M) and Down Sample (F) are the images obtained by down-sampling the moving and fixed images. We cascade the Down Sample (M) and Down Sample (F) into the low-scale registration network, that is, the blue Base Module in Figure 2D. Then, the low-scale image pairs are registered by the progressive registration strategy as proposed in Section Progressive registration strategy. We denote the final output of the low-scale network as flow-low scale. Then, the flow-low scale is upsampled two times and each pixel value is enlarged to two times the original value to obtain the flow-up sample, as formulated in Equation (8), where *Up*_2_ represents the double upsampling,


(8) $$\begin{array}{l} Flow_{Up\;Sample}=Up_{2}\left(Flow_{Low\;Scale}\right)\times 2 \end{array}$$

### Loss function design

The proposed loss function consists of similarity loss and smooth loss. Specifically, the similarity loss is utilized to restrict the alignment between the warped moving image and its corresponding fixed image. We employ the local normalized cross-correlation loss (*L*_*LNCC*_) as the similarity loss. The smooth loss is utilized to prevent excessive deformation. We utilize spatial gradient regularization as the smoothness loss (*L*_*smooth*_) of the displacement vector field.

We use the function G to indicate the shared weight module, whose input is the registration image pair, and the output is the three-channel displacement vector field (Flow). During the training process, assuming that the number of iterations is *n* (*n* > 0), then, the smooth loss of *Flow*^(*n*)^ can be formulated in Equation (9),


(9) $$\begin{array}{r} Flow^{\left(n\right)}=G\left(Warped^{\left(0,\ldots ,n-1\right)},F\;\right),\\ L_{smooth}\left(Flow^{\left(n\right)}\right)=\underset{p\in \Omega }{\sum }∥\nabla Flow^{\left(n\right)}\left(p\right){∥}^{2}. \end{array}$$

According to Section Elaboration of strategy, we conduct the AF Module to integrate *Flow*^(0)^, …, *Flow*^(*n*)^ into the total deformation field *Flow*^(0, …, *n*)^ and input it to the STN module together with the moving image to obtain the registration result *Warped*^(0, …, *n*)^ of the nth iteration, as shown in Equation (10),


(10) $$\begin{array}{l} Warped^{\left(0,\ldots ,n\right)}=STN\left(M,Flow^{\left(0,\ldots ,n-1\right)}\right) \end{array}$$

The similarity loss between the *Warped*^(0, …, *n*)^ and fixed image is calculated by Equation (11),


(11) $$\begin{array}{l} L_{Similarity}=L_{LNCC}\left(Warped^{\left(0,\ldots ,n\right)},F\right). \end{array}$$

Therefore, the objective function of the nth iteration registration can be expressed as Equation (12),


(12) $$\begin{array}{l} Loss={-L}_{LNCC}\left(Warped^{\left(0,\ldots ,n\right)},F\right)+\lambda^{*}L_{smooth}\left(Flow^{\left(n\right)}\right) \end{array}$$

where λ is the weight parameter that controls the proportion of *L*_*smooth*_ in the total loss function. The objective function calculated by Equation (12) is used for back-propagation to update the network parameters after the network completes the nth iteration,

### Elaboration of self-calibration

During the training process, the input moving item of the nth iteration is the registration result of the (n−1)th iteration. And the output warped image of the nth iteration is generated by warping the primary moving image with the integrated displacement vector field of the nth iteration. This training strategy can enable the network to learn the registration error of the previous (n−1)th iteration with the displacement vector field calibration of the nth output, and we call it the self-calibration capability of the network. Additionally, since the final warped image is obtained by interpolating the primary image once with the integrated displacement field of the nth iteration, the accumulation of warping errors caused by multi-stage cascaded networks could be avoided.

## Experiments configurations

### Datasets

We conduct a series of experiments on three datasets to verify the effectiveness of our proposed method, including one private dataset of the mouse brain and two public datasets of human brain.

The mouse brain dataset contains 21 mouse brain images and their corresponding segmentation labels, each image is submicron high-resolution multi-channel CT acquired by fMOST. The segmentation label for each image is divided into hypothalamus (HY), caudoputamen (CP), midbrain (MB), hippocampal formation (HPF), cerebral cortex (CTX), cerebellar cortex (CBX), and ventricle, where HPF and CP are divided into the left and right brain regions. We perform standard preprocessing for all images, including spatial normalization and intensity normalization. Specifically, all brains are pre-aligned to the standard mean template brain of Allen CCF with the RPM (Chui et al., 2003) algorithm, and all images are sampled from the original size 568 × 320 × 456 to 192 × 160 × 192, as well as rescale pixel values to 0–255. We randomly select 17 brains for training and the remaining four brains for testing.

The human brain datasets utilized in the experiments are LPBA40 (Shattuck et al., 2008) and OASIS-TRT. The LPBA40 contains 40 MRI images, each comes with a segmentation ground truth of 56 anatomical structures. We randomly selected 34 images as the training set and six images as the test set. The OASIS-TRT dataset is a subset of Mindboggle101 (Klein and Tourville, 2012). It contains 20 MRI images, each comes with a segmentation ground truth of 107 anatomical structures. We merge them into the same 16 anatomical segmentation structures referring to the labels utilized in VoxelMorph. We also implement standard preprocessing for all images of two human brain datasets, one reference image is randomly selected from the LPBA40 dataset, then, all human brains are pre-aligned to this reference image. The pre-alignment operation mainly includes affine registration and intensity normalization.

### Comparison settings

To demonstrate the effectiveness of our method, we compare the proposed algorithm with three existing widely used registration algorithms, including a traditional algorithm SyN (Avants et al., 2008) and two unsupervised learning-based algorithms, VoxelMorph (Balakrishnan et al., 2018) and VoxelMorph-diffeomorphic (Dalca et al., 2019). The traditional algorithm SyN is integrated into the publicly available advanced normalization tools (ANTs) which utilizes mutual information as a similarity measure for iterative optimization. The unsupervised learning-based algorithms VoxelMorph and VoxelMorph-diffeomorphic use LNCC and Smooth Regularization as the loss function. The window size of LNCC was set to *9* × *9* × *9*. We manually tune optimized regularization parameters of the smooth losses of two unsupervised algorithms. Additionally, a baseline method named Affine-only is set by only performing a global affine alignment.

### Implementation detail

We implement our method with PyTorch and accelerate training with NVIDIA GeForce RTX 3090. We use the ADAM optimizer (Kingma and Ba, 2014) with a learning rate of *10–4*, and the batch size is set to *1*. Our implementation includes a default of *30,000* iterations. We also train our method with different smoothness parameters λ until the network converges. We also implement VoxelMorph and VoxelMorph-diff with NVIDIA GeForce RTX 3090 and ANTs (SyN) with Inter(R) Core(TM) i7-10700K CPU@3.80GHZ.

### Evaluation metrics

We employ a dice score to quantitatively evaluate the registration accuracy. The dice score uses voxels to calculate the degree of overlap between the corresponding anatomical segmentation regions. The calculation formula of the dice score can be formulated as,


(13) $$\begin{array}{l} Dice\left(S_{W}^{k},S_{F}^{k}\right)=2^{\;*\;}\frac{S_{w}^{k}\cap S_{F}^{k}}{S_{w}^{k}\cup S_{F}^{k}} \end{array}$$

where $S_{w}^{k}$ and $S_{F}^{k}$ indicate the warped image and the fixed image corresponding to the kth anatomical region of the segmentation label. A dice score equal to *1* means that the corresponding anatomical regions overlap completely, and equal to *0* means that the corresponding anatomical regions do not overlap.

## Results and analysis

### Overall performance comparison

#### The results for mouse brains

We first verify the registration accuracy of all competing methods on the mouse brain dataset. Table 1 shows the average dice scores over all subjects and structures in the test set of mouse brain. As shown in Table 1, all the competing methods have improved the affine alignment significantly. Our proposed method shows 0.6–1.3% higher than the other existing state-of-the-art methods, which demonstrates that our proposed method can handle more complex deformation than the other methods. We also test the average registration times of all deformable registration methods. The computation time of the proposed method is about 0.5 s which is longer than the VoxelMorph and VoxelMorph-diff. This is reasonable, because we conduct multi-scale network cascade and iteration to achieve more accurate registration. Moreover, compared to the traditional method, our time-consuming is acceptable.

**Table 1:** The average dice scores (%) and average registration times (sec) of all competing methods on the mouse brain test set.

**Methods**	**Average dice score (%)**	**Average time (sec)**
Affine-Only	84.2954	N/A
SyN	86.6761	16.9177 (CPU)
VoxelMorph	87.3396	**0.1543 (GPU)**
VoxelMorph-Diff	87.3958	0.1571 (GPU)
Proposed	**87.9893**	0.5090 (GPU)

The best indicators are shown in bold.

**Figure 4:**
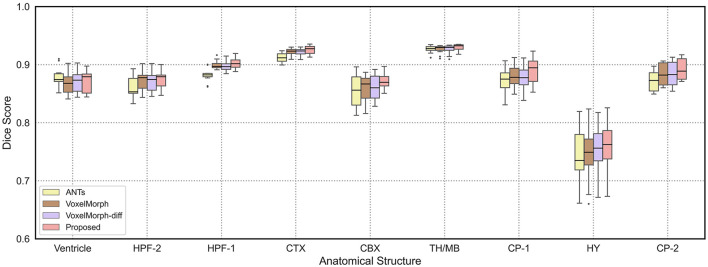
The boxplots of average dice scores of nine anatomical structures in mouse brain. We show the results of ANTs, VoxelMorph, and VoxelMorph-diff compared with our proposed method. We also mark the median values of test results with black horizontal lines.

In order to demonstrate the registration accuracy for each individual anatomical structure of mouse brain, we visualize the distributions of dice scores for the nine anatomical structures as shown in Figure 4. We can find that the median dice score of our method on each individual anatomical structure is continuously higher than the other methods which verify the advancement of our proposed method.

**Figure 5:**
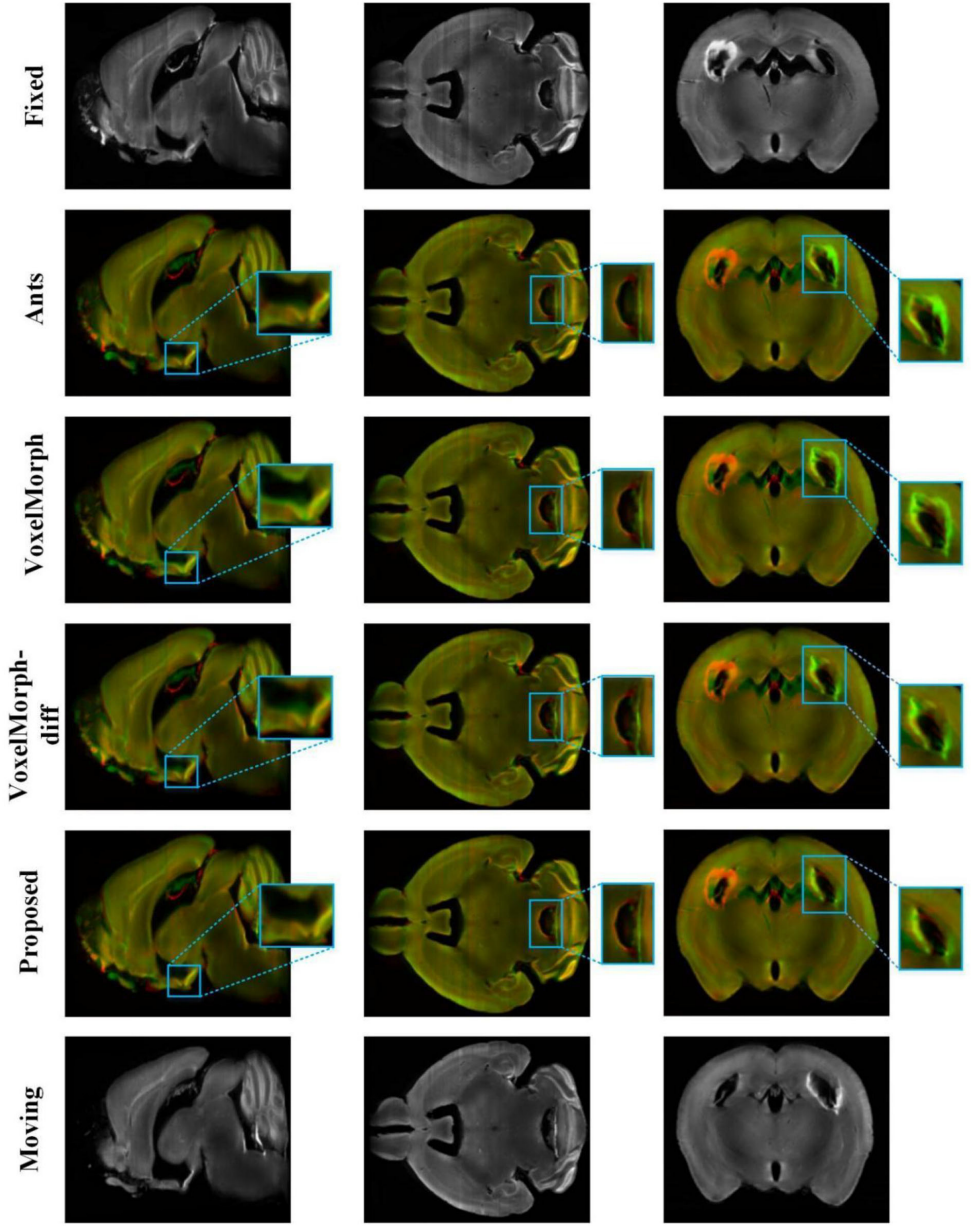
The registration results of different methods on the mouse brain test set. The 2–5 rows show 2D slices of multi-channel color blend between the registered results and the fixed image by different methods [ANTs (SyN), VoxelMorph, VoxelMorph-diff, and our proposed method] on the mouse brain test set. The red channel is the fixed image and the green channel is the different registration results. The blue box is the part we want to highlight.

Figure 5 shows the visualization results of a randomly selected image pair in the test set by different methods [ANTs (SyN), VoxelMorph, VoxelMorph-diff, and ours]. And we have performed multi-channel color blending of the registration results with the fixed image for a more intuitive observation. The visualization results are divided into three slices to display, and the spatial positions of three slices in the mouse brain are mutually independent. By comparison, it can be observed that the registration results obtained by our proposed method are more aligned with the fixed image in terms of intensity and structure than the other methods. Especially for the part in the blue box, those visually visible large non-linear deformations are well-resolved.

#### The results for human brains

We also verify the effectiveness of our proposed method on two human brain datasets, the average dice scores of all competing methods are shown in Table 2. Our proposed method shows 1.7 to 2.4% higher than the other competing methods on the LPBA40 test set and 0.8 to 3.0% higher than the other competing methods on the OASIS-TRT test set. These results demonstrate that our method is also effective for human brain registration.

**Table 2:** The average dice scores (%) of all competing methods on the LPBA40 test set and OASIS-TRT test set.

**Methods**	**Average dice score (%)**	
	**LPBA40**	**OASIS-TRT**
Affine-Only	54.679	60.155
SyN	69.339	74.249
VoxelMorph	68.624	72.041
VoxelMorph-Diff	68.884	72.159
Proposed	**71.052**	**75.100**

The bold values indicate the best indicators in the columns.

**Figure 6:**
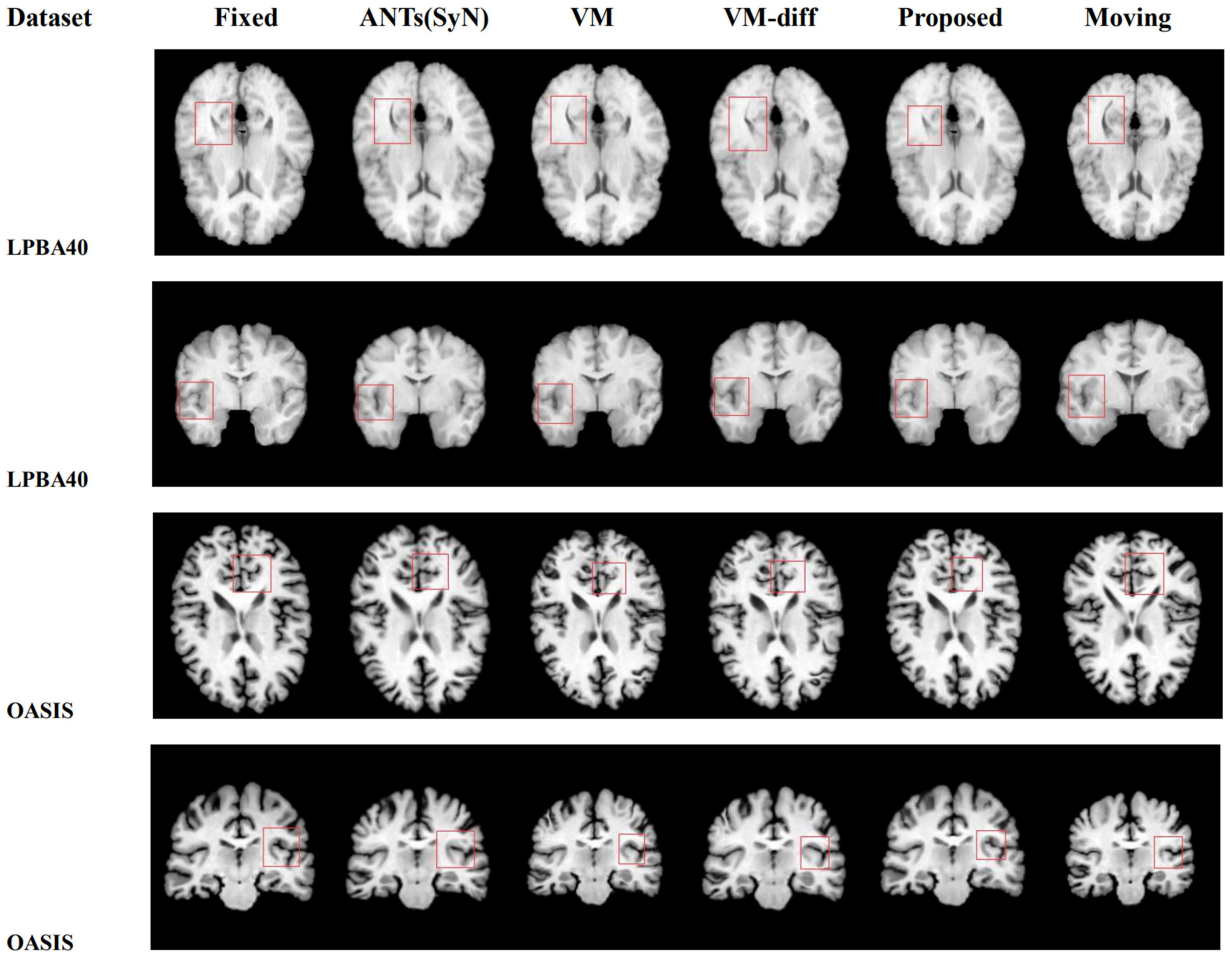
The visual 2D section of the image pair registration results randomly selected by different methods in datasets LPBA40 and OASIS-TRT.

Figure 6 is the 2D slice visualization of registration results of several randomly selected samples from the LPBA40 and OASIS-TRT test sets. It can be seen that the registration results obtained by our method match better with the fixed image in terms of brain structure and brightness than the other methods, especially for the part circled in the red box.

### Iteration parameter discussion

The hyperparameters *n_train* and *n_test* are the critical parameters in the proposed method, which denote the numbers of iterations in training and testing procedures, respectively, as described in Section Elaboration of strategy. In particular, the number of training iterations directly affects the registration accuracy. When *n_train* is set too small, the network may not be able to well learn this progressive registration strategy. In contrast, if *n_train* is set to big, the network may spend most of the time learning small deformations, which will reduce the ability of the network to handle large deformations. In order to obtain optimized hyperparameters *n_train* and *n_test*, we conduct grid search experiments of multi-scale networks on the mouse brain dataset. We increase *n_train* from *1* to *11* and *n_test* from 1 to 5, and record the dice score under each set of parameter configurations. We will demonstrate these results and discuss their effects on low-scale and original-scale networks separately.

The grad search results on low-scale network are shown in Table 3. From the observation of Table 3, we can obtain the following conclusions. First, after a column-by-column comparison, we find that the dice scores first increased and then decreased with the increase of *n_train*, this phenomenon is consistent with the conclusion of our analysis in the previous paragraph. Second, by comparing row by row, we find that the dice scores reach the best values when *n_test* is set to 3 or 4. Therefore, we choose to train the low-scale model with five iterations and utilize the results of three test iterations as the coarse registration output as well as the initial input of the original-scale network.

**Table 3:** The average dice scores (%) of low-scale network with different parameters on mouse brain test set.

	**Iteration number: 1**	**Iteration number: 2**	**Iteration number: 3**	**Iteration number: 4**	**Iteration number: 5**
Iteration number: 1	86.7183	**87.1718**	87.1433	86.9792	86.7433
Iteration number: 2	86.7895	87.2469	**87.2578**	87.1514	87.0137
Iteration number: 3	87.2113	87.6310	**87.6884**	87.6066	87.4976
Iteration number: 4	86.9859	87.5079	**87.5665**	87.5214	87.4324
Iteration number: 5	86.9678	87.5518	***87.6898***	87.6815	87.6385
Iteration number: 6	86.9066	87.4464	**87.5343**	87.5250	87.4656
Iteration number: 7	86.9492	87.5052	87.6174	**87.6225**	87.5793
Iteration number: 8	86.8978	87.4347	87.5231	**87.5337**	87.4961
Iteration number: 9	86.8077	87.4605	87.6254	**87.6455**	87.6207
Iteration number: 10	86.8908	87.4963	87.6454	**87.6827**	87.6735
Iteration number: 11	86.7796	87.2318	87.3211	**87.3327**	87.2993

The first column is the number of training iterations; the first row is the number of test iterations. The optimal value for each row is bolded and the overall optimal value is bolded and italicized.

Table 4 demonstrates the grad search results on the original-scale network, we can obtain the following conclusions. First, in the original-scale network, the changes in *n_train* and *n_test* have little effect on the registration results, this may be because most of the alignment has been done in the low-scale network, and the original-scale network only adjusts the detail areas. Second, the best dice score is achieved by setting the *n_test* to 2 or 3. By comprehensive consideration, we set *n_train* to 9 and *n_test* to 2 for the original-scale network.

**Table 4:** The average dice scores (%) of original-scale network with different parameters on mouse brain test set.

	**Iteration number: 1**	**Iteration number: 2**	**Iteration number: 3**	**Iteration number: 4**	**Iteration number: 5**
Iteration number: 1	87.8657	**87.8920**	87.8315	87.7403	87.6295
Iteration number: 2	87.9047	87.9591	**87.9573**	87.9267	87.8861
Iteration number: 3	87.9273	**87.9643**	87.9401	87.9060	87.8613
Iteration number: 4	87.9183	87.9780	**87.9875**	87.9735	87.9448
Iteration number: 5	87.9275	**87.9849**	87.9821	87.9589	87.9213
Iteration number: 6	87.8932	**87.9366**	87.9203	87.8895	87.8548
Iteration number: 7	87.9007	87.9549	**87.9687**	87.9573	87.9361
Iteration number: 8	87.8755	**87.9249**	87.9211	87.9020	87.8730
Iteration number: 9	87.9221	***87.9893***	87.9867	87.9643	87.9374
Iteration number: 10	87.8773	**87.9243**	87.9237	87.9002	87.8728
Iteration number: 11	87.9021	87.9509	**87.9542**	87.9376	87.9093

The first column is the number of training iterations, the first row is the number of test iterations. The optimal value for each row is bolded and the overall optimal value is bolded and italicized.

### Ablation study

The progressive registration strategy and hierarchical registration strategy are two key strategies proposed in our registration method. In order to verify the effectiveness of the individual strategy, we construct two comparing methods by only using the progressive registration strategy and only using the hierarchical registration strategy, which is named as progressive-only and hierarchical-only. Specifically, the hierarchical-only method is constructed by setting *n_train* and *n_test* to 1 in both low-scale and original-scale networks, and the progressive-only method is constructed by removing the low-scale network and training the original-scale network with progressive registration strategy. We refer to the optimal number of training iterations for a single scale in Table 3 and set the number of training iterations to five for the progressive-only method. The experimental results are shown in Table 5. It can be seen that each strategy in our proposed method improves the registration accuracy compared with the baseline methods, and the contribution of the progressive registration strategy to our method is greater than that of the hierarchy registration strategy at the same time. The lack of any strategy in our method will result in a decrease in registration accuracy, which reflects the effectiveness of our proposed strategies.

**Table 5:** The results of ablation experiments on the mouse brain dataset.

**Methods**		**Average dice score (%)**				
ANTs(SyN)		86.6761				
VoxelMorph		87.3396				
VoxelMorph-Diff		87.3958				
**Hierarchical-Only**	**Progressive-Only**	**Average dice score (%)**				
√		87.5124				
	√	I_N: 1	I_N: 2	I_N: 3	I_N: 4	I_N: 5
		86.797	87.582	87.806	87.859	87.870
√	√	**87.9893**				

The hierarchical-only and progressive-only stand for the two constructed competing methods, the last row indicates the proposed method contained both progressive and hierarchical registration strategies. I_N means the number of test iterations. The bold values indicate the best indicators.

### Dynamic datasets augmentation

The proposed method implicitly implements the DSS strategy (a data augmentation strategy) in DeepRS (He et al., 2020). We can dynamically augment the dataset by changing the number of training iterations. When the number of training iterations is increased once, the training dataset is implicitly doubled. We conduct an experiment to verify the inference, the results are shown in Table 6. A number of iterations of one mean that no data augmentation is performed, and this is set to the baseline. We can observe that the average dice scores obtained with other iteration numbers are all higher than the baseline, which proves the effectiveness of our implicitly dynamic augmentation strategy.

**Table 6:** Study on dynamic data augmentation at different scales.

**Training iteration number**	**Average dice score (%)**	
	**Low scale**	**Original scale**
Iteration number: 1	86.7183	87.8657
Iteration number: 2	86.7895	87.9047
Iteration number: 3	**87.2113**	87.9273
Iteration number: 4	86.9859	87.9183
Iteration number: 5	86.9678	**87.9275**
Iteration number: 6	86.9066	87.8932
Iteration number: 7	86.9492	87.9007
Iteration number: 8	86.8978	87.8755
Iteration number: 9	86.8077	87.9221
Iteration number: 10	86.8908	87.8773
Iteration number: 11	86.7796	87.9021

The number of test iterations in this table is set to 1. The bold values indicate the best indicators in the columns.

### The self-calibration capability verification

To verify the effectiveness of the self-calibration capability of the progressive registration strategy, we compare the proposed method with a conventional recurrent cascade network. These methods are trained with the same number of iterations, and we only perform the progressive registration strategy in our proposed method to eliminate the effect of the hierarchical registration strategy.

We compare these two methods with other competing methods in Table 7. After comparison, it can be concluded that the average dice score of our method on the mouse brain test set is higher than the conventional recurrent cascade network with information loss. However, it can also be seen from Table 7 that the conventional recurrent cascade network with information loss can, indeed, improve registration accuracy compared with other competing methods. The information loss of the warped moving image is caused by the recursive cascade network that interpolates and deforms the moving image many times. As the number of iterations increases, the details of the images to be registered in the input sub-network are lost more and more seriously. So that the subsequent registration sub-network cannot repair and make good use of the detailed information of the image.

**Table 7:** The average dice scores (%) between the conventional recursive cascade method with information loss and our proposed method on the mouse brain test set.

**Methods**	**Average dice score (%)**
Affine-Only	84.2954
SyN	86.6761
VoxelMorph	87.3396
VoxelMorph-Diff	87.3958
Recursive cascade method	87.6885
Proposed	**87.8700**

We also compare these two methods with other competing methods. The bold values indicate the best indicators.

**Figure 7:**
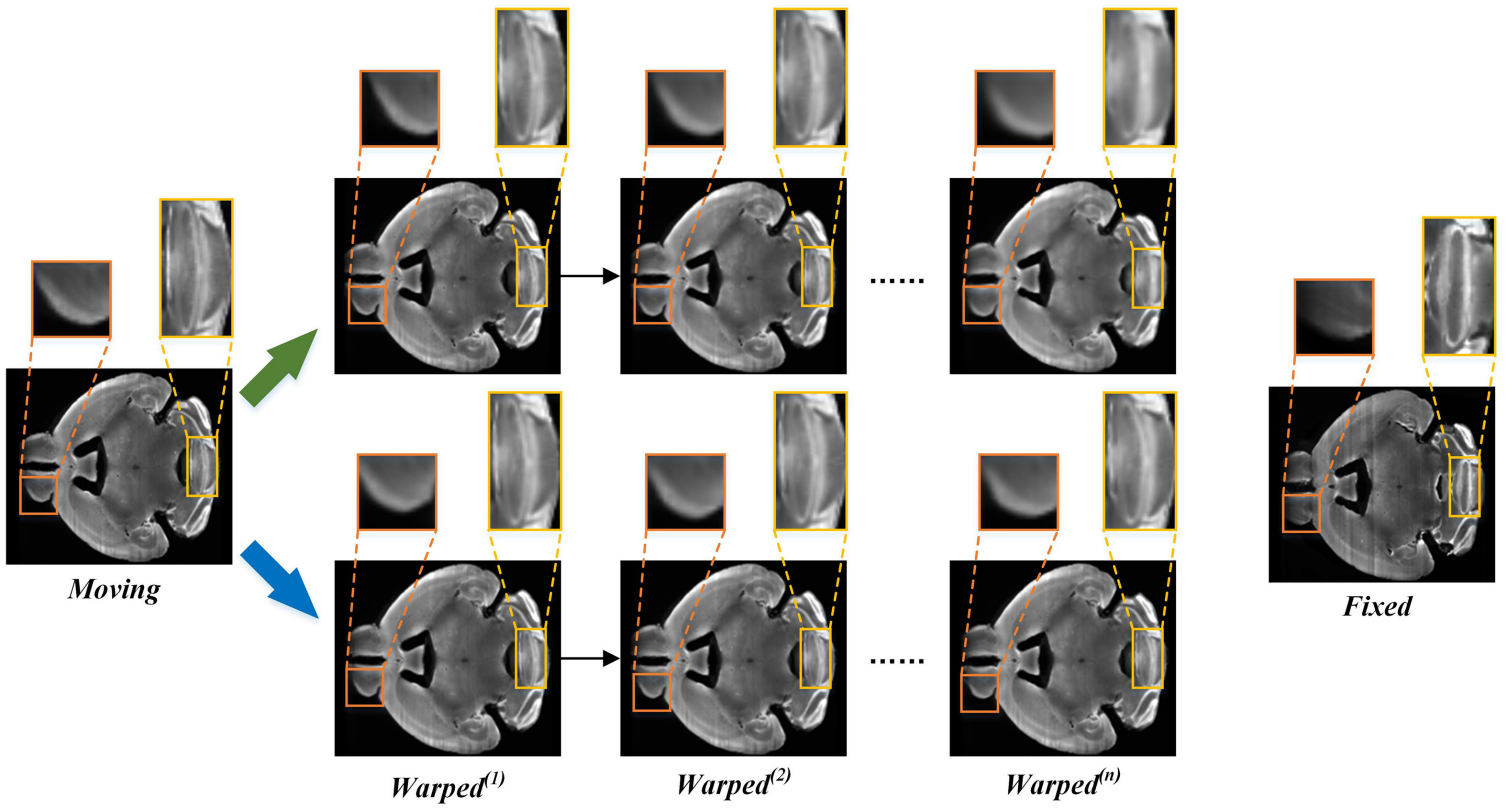
The intermediate registration results generated by our method and conventional cascade registration methods with information loss in the iterative process. The green arrow points to the iterative registration process of the conventional recursive cascade method with information loss, and the blue arrow points to the iterative registration process of our method.

In order to intuitively show the process of information loss in the conventional recurrent cascade network, we plot some intermediate results of registration in Figure 7. The direction of the green arrow is the visualization result of the recursive cascade network with information loss, and the blue arrow is the visualization result of our proposed method. By comparing the parts in the orange and yellow boxes, we can conclude that as the number of iterations increases, the edge details of the conventional recursive cascade method warped moving images become more and more blurred. However, as the number of iterations increases, the warped moving image generated by our method in the registration process can always maintain a clear boundary.

**Figure 8:**
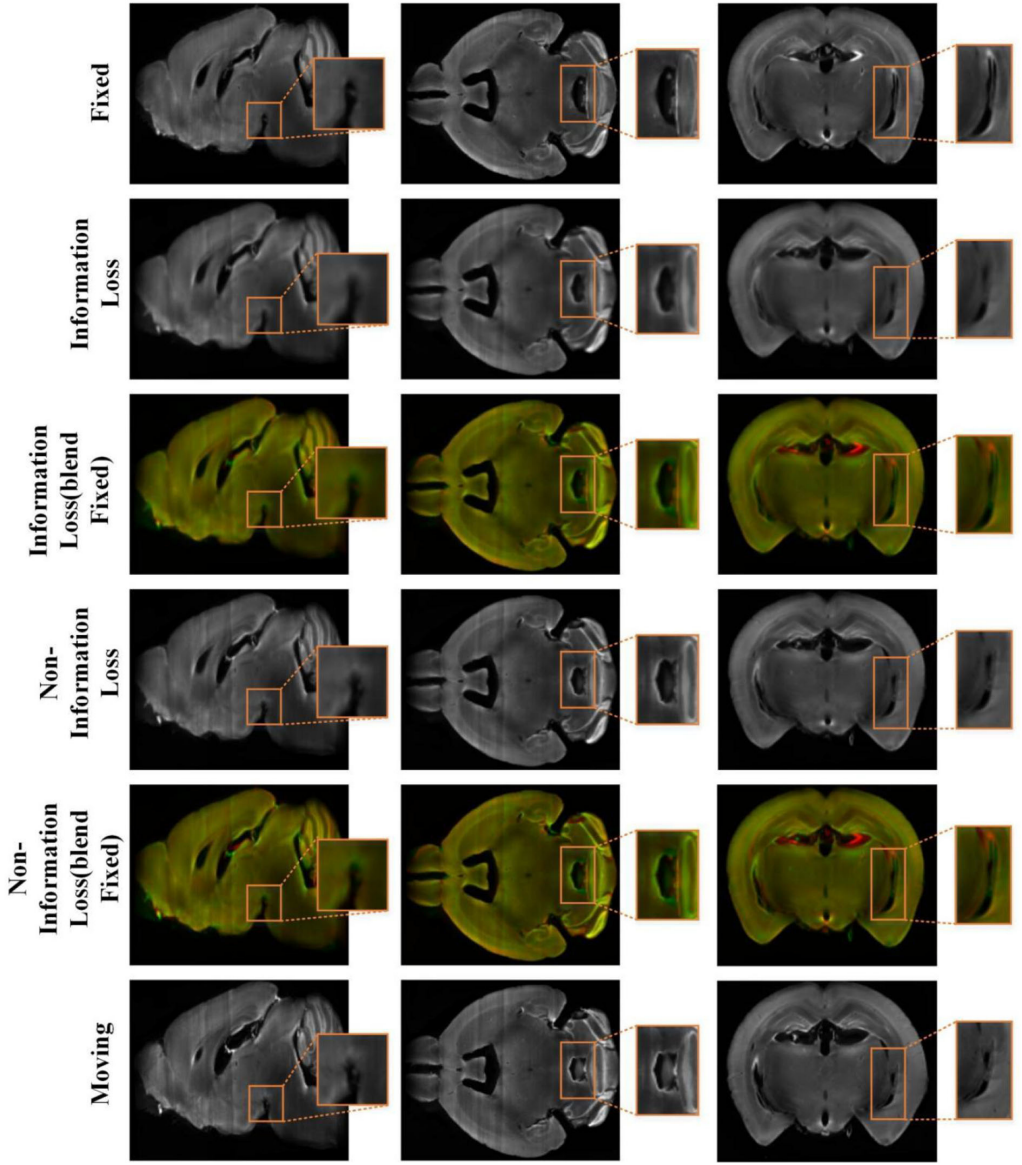
The registration results of the conventional cascade registration method with information loss and our method on the mouse brain test set. And the visualization of 2D slices multi-channel color blend between the two registered results and the fixed image. The red channel is the fixed image and the green channel is the different registration results. The orange box is the part we want to highlight.

Figure 8 shows the visualization results of randomly selected instance image pairs (17,545–17,781) for registration between our method and the conventional recursive cascade registration method in the mouse brain test set. Figure 8 also shows the visualization of 2D slices multi-channel color blend between the two registered results and the fixed image. The visualization results are divided into three sections to display. The spatial positions of the slices in the mouse brain are independent of each other. By comparing these visualization slices, especially the part in the orange box, it can be seen that the registration results obtained by our method have clear boundaries and details compared with those obtained by the conventional recursive cascade method. The registration results obtained by the conventional recursive cascade method have certain losses in terms of intensity and details. And the registration results obtained by our proposed method are more aligned with the fixed image in both intensity and structure than the conventional recursive cascade method.

## Conclusion

In this article, we have proposed a progressive network based on deep self-calibration for deformable biomedical image registration, which leverages the idea of progressive cascaded networks to handle the large non-linear deformation and reduce the problem of information loss existing in the existing progressive registration. The proposed method consisted of two registration strategies, the progressive registration strategy was designed to reduce the cascaded information loss, and the hierarchical registration strategy was conducted to achieve a fast and coarse-to-fine registration. In addition, our proposed progressive registration strategy could generate abundant training data during the training procedure, which has a significant advantage for biological image analysis with fewer available data. However, our proposed method focuses on the mono-modal registration task, and the multi-modal registration still remains challenging in biomedical deformable registration. Generally, the modal disparity of cross-modal images is large, and it is difficult to obtain satisfactory results in a one-shot registration. Therefore, further work will be done on our proposed progressive registration network to meet the cross-modal registration.

## Data availability statement

The original contributions presented in the study are included in the article/Supplementary material, further inquiries can be directed to the corresponding author.

## Author contributions

RS, JW, and LQ contributed to the conception and design of the proposed registration method. RS completed the core experiments and the writing of the first draft. JW guided the experiment and improved the writing of the article. LQ provided constructive comments and determined the final draft of the article. LO and YM helped complete the comparative experiment section. All authors contributed to the drafting of the manuscript. All authors contributed to the article and approved the submitted version.

## Funding

This research was funded by the National Natural Science Foundation of China (61871411), the University Synergy Innovation Program of Anhui Province (GXXT-2021-001), the Natural Science Foundation of Education Department of Anhui Province (KJ2021A0017), and the National Natural Science Foundation of China (Nos. 62271003 and 62201008).

## Conflict of interest

The authors declare that the research was conducted in the absence of any commercial or financial relationships that could be construed as a potential conflict of interest.

## Publisher's note

All claims expressed in this article are solely those of the authors and do not necessarily represent those of their affiliated organizations, or those of the publisher, the editors and the reviewers. Any product that may be evaluated in this article, or claim that may be made by its manufacturer, is not guaranteed or endorsed by the publisher.
