# Supplementary material for: Progressive 3D biomedical image registration network based on deep self-calibration
Source: Front Neuroinform. 2022 Sep 21;16:932879. doi: 10.3389/fninf.2022.932879 (PMC9532554; doi:10.3389/fninf.2022.932879)
Supplement: Supplementary file 1 [file Data_Sheet_1.PDF]

VALIDATION ON HUMAN BRAIN MRI DATASETS

In addition to the optical brain images, our method can also be applied to register MRI datasets. We tested the performance of our method by using two public MRI datasets. One of them is LPBA40. This dataset contains 40 scans, each comes with a segmentation ground truth of 56 anatomical structures. We select one scan as the reference, use 34 scans as the training sets and treat the rest 6 scans as the test sets. The other is OASIS-TRT, which is the subset of Mindboggle101. This dataset contains 20 scans, each comes with a segmentation ground truth of 107 anatomical structures. We divided it into the same 16 anatomical segmentation structures as in VoxelMorph . We use the standard preprocessing steps: affine pre-registration of all images in LPBA40 with OASIS-TRT onto the first image of the LPBA40 dataset and normalize the intensity.

As shown in Figure 1, we obtain models with different parameters by setting different training iterations and use these them to test on the LPBA40 test set. As shown in Figure 1A, in the testing phase of the network, with the increase of the number of iterations of the models with different parameters, the dice score reaches the highest when the model is iterated for 21 times. As shown in Figure 1B, in the testing phase of the network, with the increase of the number of iterations of the models with different parameters, the dice score reaches the highest when the model is iterated for 30 times. Since the model trained with the number of training iterations set to 1 or 2 does not fully learn the progressive registration strategy, it is not drawn in the figure. And also from Figure 1, we can see that with the increase of the number of iterations of the training setting, the best accuracy achieved by the models at both scales shows a trend of first increasing and then decreasing. Finally, we choose to cascade the low-scale network with 7 iterations of training, 21 test iterations and the original-scale network with 7 training iterations and 30 test iterations as the final model.

We utilize the final model to compare with the baseline methods on the LPBA40 test set and the OASIS-TRT test set, and the results are shown in Table 1. Our proposed method shows 1.7 to 2.4 percentage higher than the baseline methods on the LPBA40 test set and 0.8 to 3.0 percentage higher than the baseline methods on the OASIS-TRT test set. Furthermore, we predicted the LPBA40 test set and computed the dice scores of 56 brain regions, and predicted the OASIS-TRT test set and computed the dice scores of 16 brain regions. The results are shown as a box plot in Figure 2A-D. It can be seen that our method outperforms the baseline methods in most brain regions on both test sets. Figure 2E is a 2D slice visualization of the registration results of our randomly selected pair of images under the LPBA40 and OASIS-TRT test sets. It can be seen that, especially for the part circled in the red box, the registration results obtained by our method match better with the fixed image in terms of brain structure and brightness than the other methods.

TABLES AND FIGURES

Table 1. The average dice score (%) of different methods on the LPBA40 test set and the average dice score (%) on the OASIS-TRT test set

Methods	Average Dice Score(%)	
	LPBA40	OASIS-TRT
Affine-only	54.679	60.155
SyN	69.339	74.249
VoxelMorph	68.624	72.041
VoxelMorph-diff	68.884	72.159
Proposed	71.052	75.100

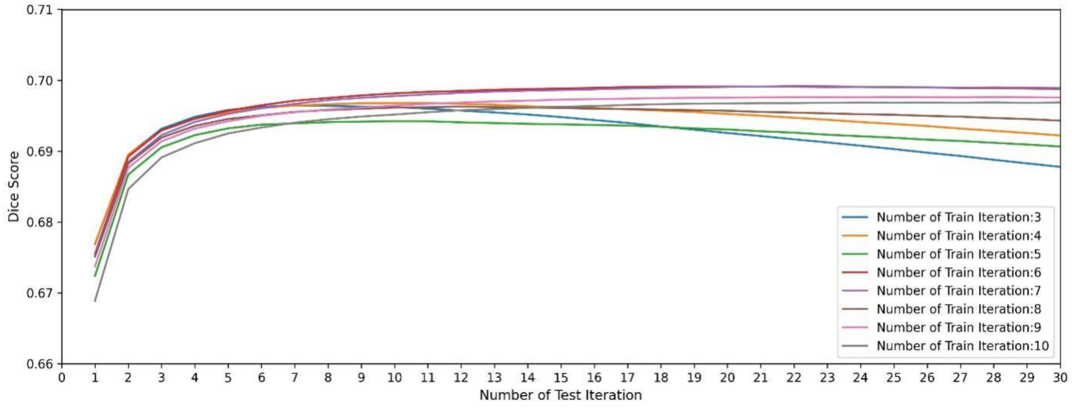

(A)

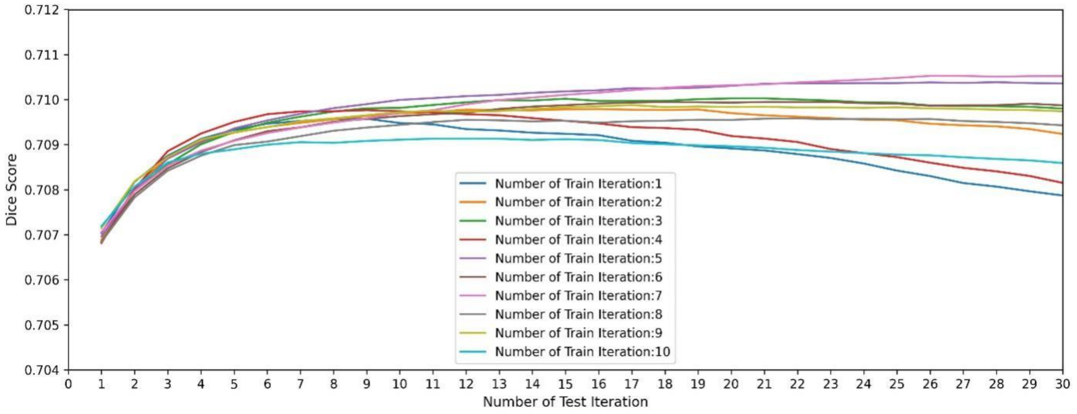

(B)

Figure 1 (**A)** Average dice score (%) of low-scale networks with different parameters on LPBA40 test set. Different curves indicate different model training iterations. (B) Average dice score (%) of original-scale networks with different parameters on LPBA40 test set. Different curves indicate different model training iterations.

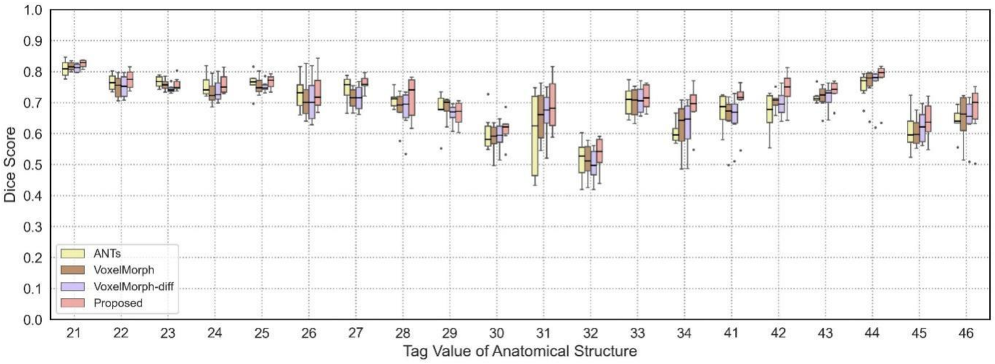
Dice Score
Tag Value of Anatomical Structure
ANTs
VoxelMorph
VoxelMorph-diff
Proposed

(A)

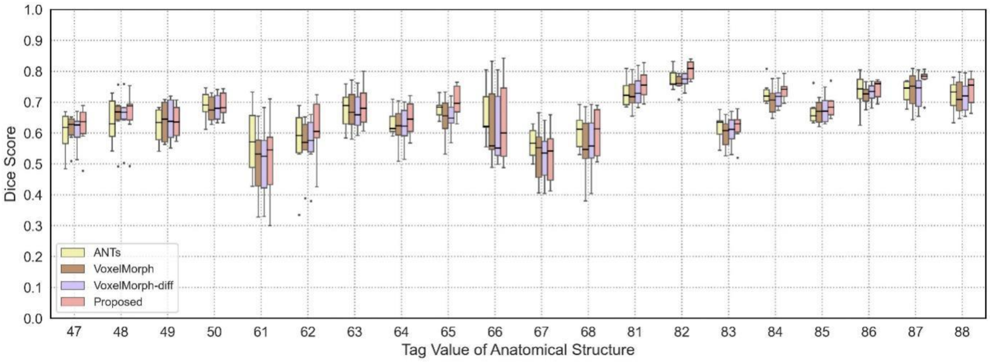
Dice Score
Tag Value of Anatomical Structure
ANTs
VoxelMorph
VoxelMorph-diff
Proposed

(B)

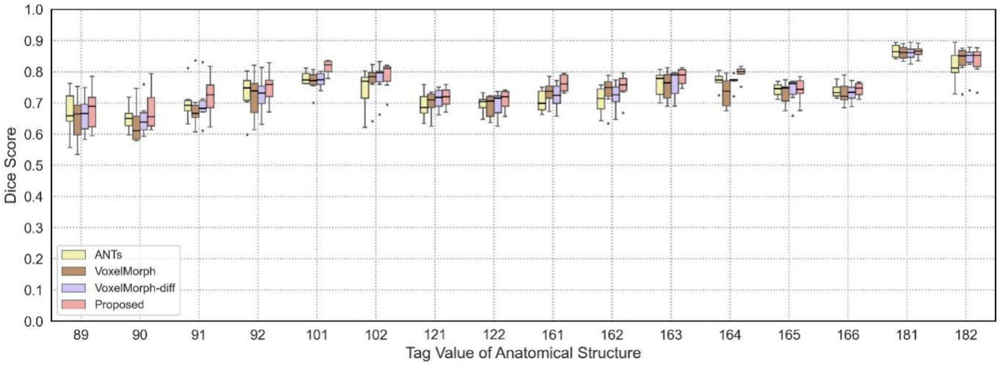
Dice Score
Tag Value of Anatomical Structure
ANTs
VoxelMorph
VoxelMorph-diff
Proposed

(C)

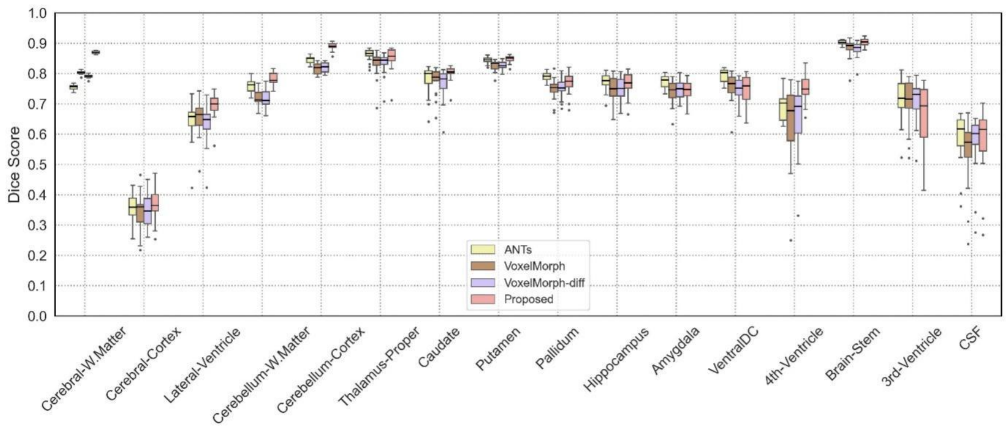

(**D**)

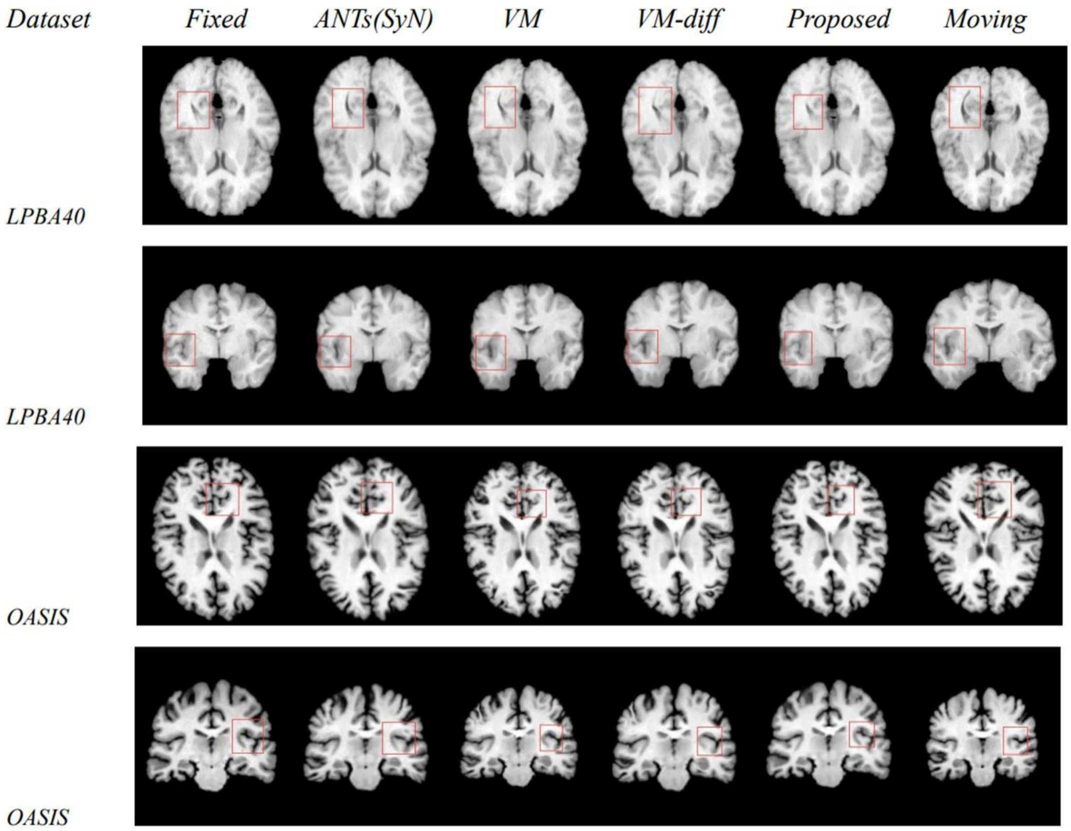

(E)

Figure 2 **(A)**, **(B)** and **(C)** show the boxplot of the dice score of 56 anatomical structures for each pair of registered images in LPBA40 test set. **(D)** shows the boxplot of the dice score of 16 anatomical structures for each pair of registered images in OASIS-TRT test set. **(E)** shows the visual 2D section of the example image pairs' registration results randomly selected by different methods in datasets LPBA40 and OASIS-TRT.
